# Assistive Arm-Exoskeleton Control Based on Human Muscular Manipulability

**DOI:** 10.3389/fnbot.2019.00030

**Published:** 2019-05-29

**Authors:** Tadej Petrič, Luka Peternel, Jun Morimoto, Jan Babič

**Affiliations:** ^1^Laboratory for Neuromechanics and Biorobotics, Department for Automatics, Biocybernetics and Robotics, Jožef Stean Institute, Ljubljana, Slovenia; ^2^Department of Cognitive Robotics, Delft University of Technology, Delft, Netherlands; ^3^Department of Brain-Robot Interface, ATR Computational Neuroscience Labs, Kyoto, Japan

**Keywords:** exoskeleton control, manipulability analysis, robot assistance, arm exoskeleton, human-robot interaction

## Abstract

This paper introduces a novel control framework for an arm exoskeleton that takes into account force of the human arm. In contrast to the conventional exoskeleton controllers where the assistance is provided without considering the human arm biomechanical force manipulability properties, we propose a control approach based on the arm muscular manipulability. The proposed control framework essentially reshapes the anisotropic force manipulability into the endpoint force manipulability that is invariant with respect to the direction in the entire workspace of the arm. This allows users of the exoskeleton to perform tasks effectively in the whole range of the workspace, even in areas that are normally unsuitable due to the low force manipulability of the human arm. We evaluated the proposed control framework with real robot experiments where subjects wearing an arm exoskeleton were asked to move a weight between several locations. The results show that the proposed control framework does not affect the normal movement behavior of the users while effectively reduces user effort in the area of low manipulability. Particularly, the proposed approach augments the human arm force manipulability to execute tasks equally well in the entire workspace of the arm.

## 1. Introduction

To date, many exoskeleton systems have been designed and controlled to either assist or resist the human motion depending on the application type. These systems enclose either a larger part of the human body or just individual joints. Most of the control methods here are focused on augmenting the effectiveness of the users in terms of joint motion or joint torque. To enable safe interaction between the exoskeleton and the user, a common approach is to use an impedance or admittance controller (Tsagarakis and Caldwell, 2003; Marchal-Crespo and Reinkensmeyer, 2009), where the interaction forces are controlled through a mass-spring-damper system (Hogan, 1985). Contrarily, in power augmentation tasks the exoskeleton needs to provide additional joint torques to augment the existing body capabilities. Here movement intentions of the user and corresponding joint torques are obtained by either direct force/torques measurements (Pratt et al., 2004; Kong and Jeon, 2006) or muscle activity measurements (Petrič et al., 2011). The most common approach for measuring the muscle activity in exoskeleton control methods is the use of electromyography (EMG) (Fleischer et al., 2005). Different methods can be used to map muscle activities into the joint torques, such as biomechanical models (Rosen et al., 2001; Fleischer and Hommel, 2008), proportional mapping (Ferris et al., 2006; Lenzi et al., 2012; Koller et al., 2017; Toxiri et al., 2018) or machine learning algorithms (Peternel et al., 2016).

Many of these power augmentation methods are design to amplify the force evenly regardless of the limb configuration and the desired direction of movement. However, the force capability of the user's limb endpoint is heavily dependent on its current configuration and the direction of movement. A common approach to evaluate the biomechanical performance of a limb endpoint is to use manipulability measure that was initially derived for analysis of anthropomorphic robots (Yoshikawa, 1985). Manipulability is a measure that describes the relationship between joints and limb endpoint with respect to velocity (Yoshikawa, 1985; Vahrenkamp et al., 2012), acceleration (Chiacchio and Concilio, 1998; Yokokohji et al., 2009) or force (Bicchi et al., 1997; Gravagne and Walker, 2002; Tanaka et al., 2015). These measures are used to evaluate the effects of instantaneous variation in joints on the variation at the endpoint, and is usually represented by a spheroid around the endpoint. The distance from the endpoint to the spheroid surface in a given direction represents the maximal feasible velocity, acceleration or force capacity in that direction.

A few studies explored how to exploit manipulability measures for human motion augmentation. In a study (Petrič et al., 2016), we proposed a control approach that compensates the anisotropic property of the kinematic manipulability related to the human arm. Similarly, in Shen et al. (2017), the control approach was improved by incorporating endpoint loading conditions into the modified manipulability models. In Kim et al. (2010), the dynamic manipulability was used to generate an energy efficient gait pattern. However, these control methods (Kim et al., 2010; Petrič et al., 2016; Shen et al., 2017) were only based on human limb kinematics without considering also biomechanical specifics of human arms, i.e., muscles. In contrast to typical robotic actuators with gears and motors, the human joints are actuated by sets of antagonistically coupled muscles. The force generation capacity is configuration dependent and the relationship between the muscle forces and joint torques is nonlinear.

Many studies in human biomechanics already thoroughly analyzed the relationship between the joint torque and joint angle in lower limbs (Anderson et al., 2007), upper limbs (Leedham and Dowling, 1995; Kentel et al., 2011) and whole body (Millard et al., 2013). Analysis of the human force manipulability was introduced in Jacquier-Bret et al. (2012); Yu and Liang (2012), but without consideration of the specifics of the human actuators. Nevertheless, a detailed study which would address the relationship between joint torques and forces at the limb endpoint in terms of movement or force capabilities was still missing. The quantitative evaluation of force generation at the endpoint was reported in Sasaki et al. (2010), where manipulability models were developed using the human joint torque characteristic. To properly account for the effect of specific characteristics of human joint on endpoint manipulability, a study recently derived a manipulability model of the endpoint via human muscle forces (Ohta et al., 2014).

To address the limitations of the control method in Kim et al. (2010); Petrič et al. (2016); Shen et al. (2017), we propose a novel control method for an upper body assistive device which takes into account the human arm muscular force manipulability (Ohta et al., 2014). The proposed method derives from biomechanical studies to account for configuration dependent force capabilities of the human arm and selectively augments the user endpoint force capabilities based on the current arm configuration and motion direction. As a result, the exoskeleton provides more support to the arm in configurations and directions of motion where the force manipulability is smaller, and vice versa, less support to arm in configurations and directions of motion where the force manipulability is high. As a consequence, the proposed exoskeleton controller effectively maintains a spherical endpoint force manipulability of the human arm in the entire workspace.

To analyze the effects of the controller on the human motion, we hypothesize that the proposed control approach will reduce the human effort without diverting from the normal unassisted motion trajectory. To validate the proposed approach and hypothesis, we performed an experimental study on nine subjects, who were wearing a two degrees-of-freedom (DoF) arm exoskeleton. Their task was to move a 4 kg weight between two different target locations. We used a surface EMG to measure the effort of each subject during the task execution.

A preliminary study was presented at 2017 IEEE International Conference on Robotics and Automation (Goljat et al., 2017), where the method was introduced and evaluated only on a single subject. The specific contributions of this paper are: an extended evaluation based on data from nine naive subjects supported with a statistical analysis, an extended method formulation with a more in-depth explanation, a more thorough overview of the related work, and an additional discussion of novel results.

## 2. Force Manipulability

Manipulability is defined by the kinematics of the mechanism, where the joint angle variations are propagated into the endpoint variations (Yoshikawa, 1985). The arm manipulability can be expressed with an ellipsoid around the endpoint whose radius represents the capacity of movement in different directions of Cartesian space. Orthogonally to the manipulability ellipsoid is the force ellipsoid, whose radius represents the capacity of exerting a force in different directions. The direction of the largest force capacity is also the direction where the robot is the least sensitive to the actuator errors (Gravagne and Walker, 2002).

**Figure 1:**
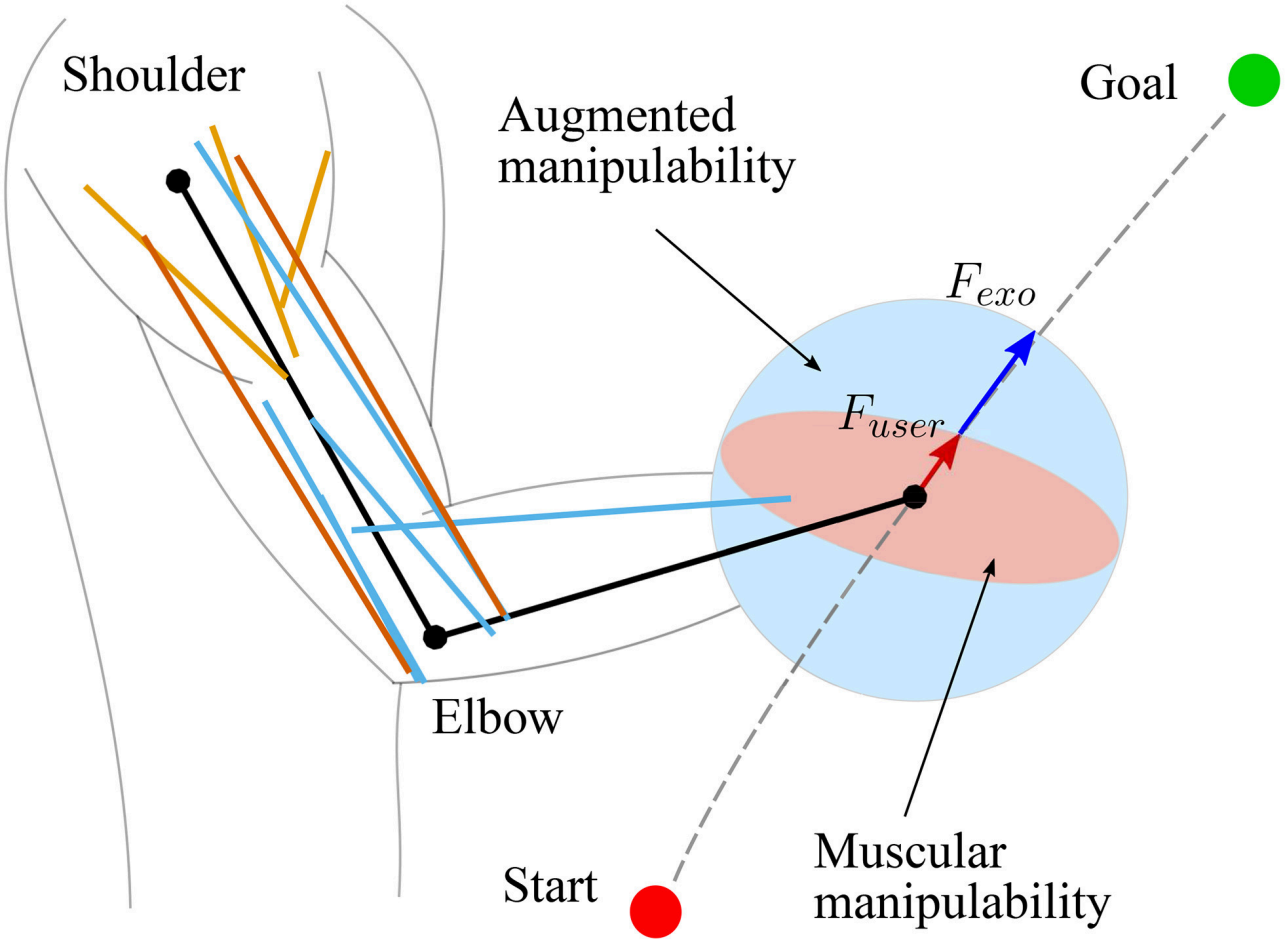
Illustrated representation of proposed method using a muscular manipulability model (Goljat et al., 2017). The human arm is modeled as a two-segment rigid-body mechanism that is actuated by ten muscles: three shoulder muscle (sternal and clavicular part of Pectoralis major and Deltoid muscle), two bi-articular muscles (Triceps long head and Biceps short head), and five elbow muscles (Triceps lateral and medial head, Biceps long head, Brachialis and Brachioradialis). The red ellipse represents the human arm muscular force manipulability, and the blue circle shows the resultant force manipulability of the combined system of human arm with assistive device.

The classic manipulability measures assume that the joints are driven by the actuators (e.g., motors) that can produce equal joint torque in both directions, independently of the configuration. However, human arm is driven by muscles, whose torque production characteristics change with the configuration of the arm. Therefore, the classic manipulability measures need to be updated to account for these properties. In literature, the models that can account for such human specifics are called muscular manipulability models (Tanaka et al., 2005; Ohta et al., 2014). In this paper, we extended the muscular manipulability model of the arm (Ohta et al., 2014) to used it for controlling an arm exoskeleton. The main goal of the control concept is to augment the human motion using the muscular manipulability model. As a result, the human arm force manipulability becomes spherical throughout the entire workspace. The conceptual idea is illustrated in Figure 1.

The following sub-sections first provide the mathematical formulation of the classical manipulability measure and then its extension toward the muscular manipulability model. For the sake of clarity, the method is explained on a planar case, where ellipsoids are reduced to ellipses. Nevertheless, the method is general and operates in the 3D space.

### 2.1. Force Manipulability

The Jacobian matrix **J** describes the relationship between the joint velocities and endpoint velocities, while **J**^−*T*^ describes the relationship between the joint torques and endpoint forces. In case of a non-redundant mechanism, eigenvalues and eigenvectors of either matrix define manipulability ellipse and force manipulability ellipse, respectively. In a general case when mechanism has redundant DoFs, the ellipse can be derived by mapping all possible variables in joint space, contained within a unit circle, into the endpoint variables in the Cartesian space. A set of all joint toque variables contained within the unit circle is described by:

(1) $${\left\|\tau \right\|}^{2}=\tau^{T}\tau \leq \text{1},$$

where **τ** is joint torque vector. In general, the transformation from the joint torques to the endpoint forces is given by:

(2) $$\begin{array}{r} \tau ={\text{J}}^{T}\left(\text{q}\right)\text{F}, \end{array}$$

where ***F*** is the endpoint Cartesian force/torque vector and ***q*** is the joint angle vector. By inserting (2) into (1) we get:

(3) $${\left\|{\text{J}}^{T}\text{F}\right\|}^{2}={\text{F}}^{T}({\text{JJ}}^{T})\text{F}\leq 1,$$

where the inner product $\text{J}{\text{J}}^{T}={\text{M}}_{v}$ is used to compute the manipulability and ${\left(\text{J}{\text{J}}^{T}\right)}^{-1}={\text{M}}_{F}$ is used to compute the force manipulability. Using singular value decomposition (SVD) of **M**,

(4) $$\text{M}=\text{U}\sum {\text{V}}^{*},$$

where **U** is a unitary matrix, **Σ** is a diagonal matrix with non-negative real numbers on the diagonal, **V** is unitary matrix, and V^*^ is the conjugate transpose of **V**, we can obtain the singular vectors, which correspond to the minor and major axes of the manipulability ellipse (Yoshikawa, 1985). Minor and major axes represent the directions in which the lower and the higher forces can be generated respectively. Even though this is a kinematic-based metric, it has still been used in several studies of human motion (Sabes and Jordan, 1997; Hara et al., 1998; Tanaka et al., 2005).

### 2.2. Muscular Force Manipulability

To account for the forces that are generated by the muscles acting on the joints, we derive the muscular manipulability measure, which describes the transformation between the muscle forces and the endpoint forces. First, the transformation of the muscle forces to the joint torques is governed by:

(5) $$\begin{array}{r} \tau ={\text{J}}_{m}^{T}\left(\text{q}\right){\text{F}}_{m}, \end{array}$$

where **J**_*m*_ is the muscle Jacobian matrix that maps muscle forces ***F***_*m*_ into joint torques. **J**_*m*_ matrix also represents the muscle moment arms for each joint. The moment arms for the extensor muscles were defined as the shortest distances between the centers of the joints and the lines connecting the origins and the insertions of the muscles. The parameters for the origin and insertion points of the muscles were selected from the literature (Wood et al., 1989). By merging the (Equations 2, 5) we get the relationship between the muscles forces and the endpoint forces as:

(6) $$\begin{array}{r} \text{F}={\text{J}}^{-T}{\text{J}}_{m}^{T}{\text{F}}_{m}. \end{array}$$

To account for the muscular activation levels we use the Hill's muscle model. The relationship between muscle forces and endpoint forces now derives into:

(7) $$\begin{array}{r} F={\text{J}}^{-T}{\text{J}}_{m}^{T}{\text{F}}_{h}\alpha , \end{array}$$

where *F*_*h*_ is the diagonal matrix of the Hill's muscle force equation and the muscular activation levels are bounded to ‖α‖ < 1. Note that muscle activation is greater than 0 at rest and less than 1 at max. In the same manner as in (3), by using (7) we get the expression that determines the muscular manipulability

(8) $$\begin{array}{r} {\text{M}}_{m}=\left({\text{J}}^{-T}{\text{J}}_{m}^{T}{\text{F}}_{h}\right){\left({\text{J}}^{-T}{\text{J}}_{m}^{T}{\text{F}}_{h}\right)}^{T}. \end{array}$$

Similarly as before, we use a singular value decomposition of **M**_*m*_ to obtain singular vectors that correspond to the minor and major axes of the muscular force manipulability ellipse.

## 3. Exoskeleton Control

In this section, we describe the proposed exoskeleton control method based on the muscular force manipulability model. The anthropometric data for the arm, muscles, muscle-tendon lengths and moment arms were obtained from Langenderfer et al. (2004); Holzbaur et al. (2005). The muscle force was modeled with Hill-type representation (Hill, 1938; Zajac, 1989) given by:

(9) $$\begin{array}{r} F_{m,i}=\left(f_{0,i}f_{l,i}f_{v,i}\alpha +F_{p,i}\right)cos\left(\phi \right), \end{array}$$

where *i* is the i-th muscle, *f*_*l*_ is the active force-length relationship, α is the activation level, ϕ is the muscle-tendon pennation angle, *f*_0_ is the optimal muscle force and *f*_*v*_ is the force-velocity relationship. We neglected the passive part since its force contribution is low due to the constant muscle activation during the motion (Jo, 2011). The normalized tendon slack lengths are also small, therefore we assume that tendons are stiff and have a negligible effect on the generated force (Zajac, 1989). Furthermore, all human arm muscles have a pennation angle smaller than 20:

(10) $$\begin{array}{r} F_{m,i}=f_{0,i}f_{l,i}f_{v,i}\alpha . \end{array}$$

**Figure 2:**
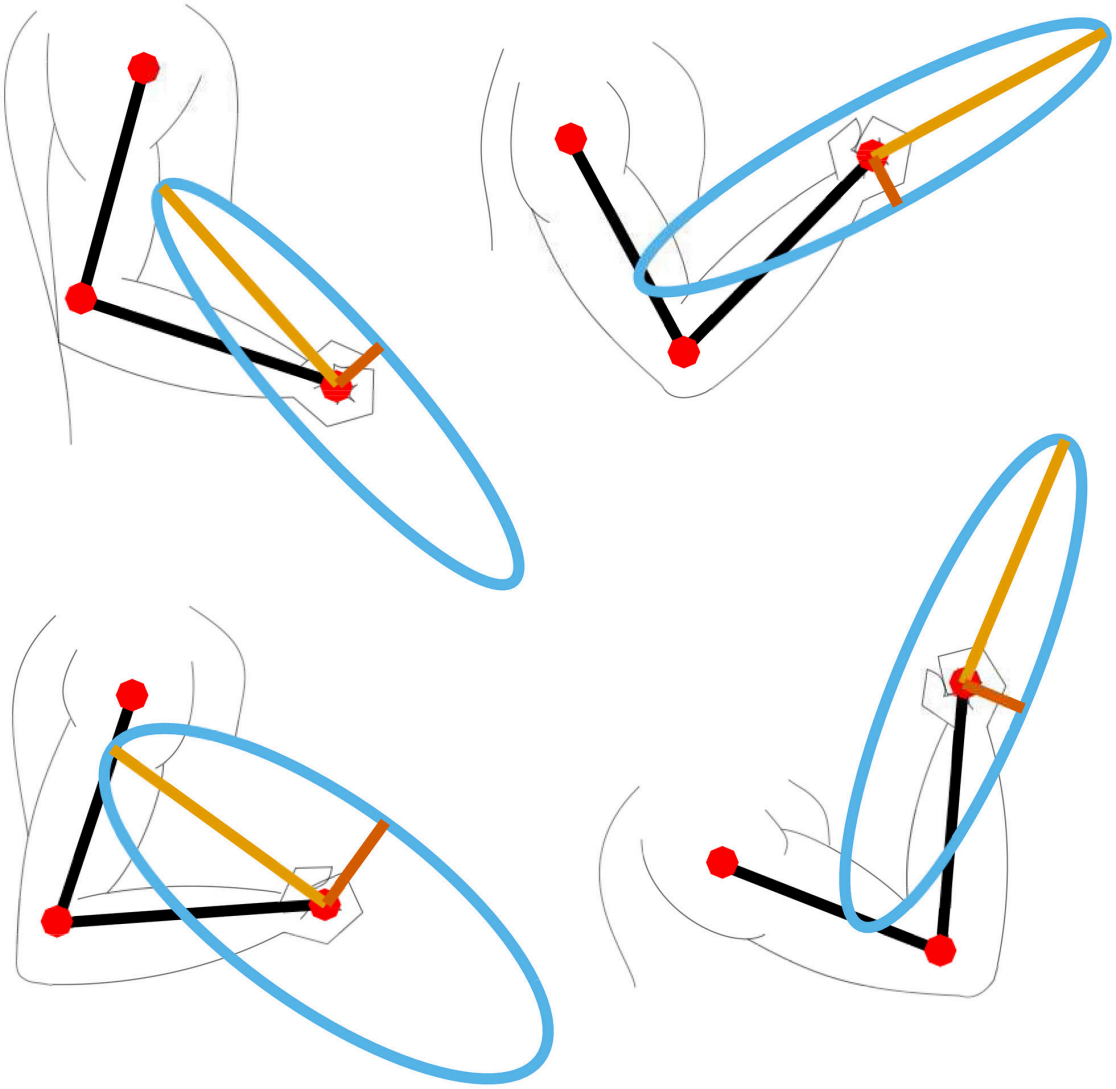
Example of four different arm configurations and their corresponding muscular force manipulability ellipses.

Here the product of the parts *f*_0, *i*_, *f*_*l, i*_ and *f*_*v, i*_ is equal to the diagonal matrix of the Hill's muscle force *F*_*h*_ used in (8). The detailed parameters of the optimal muscle length, maximal muscle force, and tendon slack can be found in Ning Lan (2002); Buchanan et al. (2004); Colacino et al. (2012). In our model we have included a total of ten muscles: three shoulder muscle (sternal and clavicular part of Pectoralis major and Deltoid muscle), two bi-articular muscles (Triceps long head and Biceps short head), and five elbow muscles (Triceps lateral and medial head, Biceps long head, Brachialis and Brachioradialis), as shown in Figure 1. To compute the muscle Jacobian **J**_*m*_ we used the parameters of muscle origins and insertions from Wood et al. (1989). By inserting the Jacobian **J**_*m*_ in (8), we computed the muscular manipulability matrix **M**_*m*_. Furthermore, by using the singular value decomposition we got the minor and major axes of the muscular manipulability. The minor axis represents the direction in which the ability to produce the endpoint force is low, while the major axis represents the direction in which the ability to produce the force is high. Examples of the muscular force manipulability ellipses for the two DoF arm model are shown in Figure 2. Note that for computing the muscular force manipulability ellipsoids we do not need to record or capture any EMG data, i.e., it si computed based on a model whose input is the configuration of the arm.

The proposed control method augments the human motion in a way that the force manipulability shape of the human arm endpoint results in a circle, i.e., human force production capacity is equal in all directions throughout the entire workspace. Note that the level of support is not discrete but varies continuously based on the calculated muscular manipulability at any sample time in online manner. To do so, the arm exoskeleton device scales the user endpoint force based on the force manipulability model. The illustration of the scaling is illustrated in Figure 1. The supporting force *F*_*e*_ that the exoskeleton produces is governed by:

(11) $$\begin{array}{r} {\text{F}}_{e}=K\left({\text{M}}_{m},{\text{F}}_{u}\right){\text{F}}_{u}, \end{array}$$

where *K*(**M**_*m*_, *F*_*u*_) is a function that is computed based on the muscular manipulability model in the direction of user's force and is defined as:

(12) $$\begin{array}{r} K\left({\text{M}}_{m},{\text{F}}_{u}\right)=\frac{\lambda_{m}}{{\hat{\text{F}}}_{u}}-1, \end{array}$$

where λ_*m*_ = max(diag(**Σ**)) is the maximal singular value of **M**_*m*_. Here ${\hat{\text{F}}}_{u}$ is the force manipulability capacity in the direction of user's force ***F***_*u*_ and is defined as:

(13) $$\begin{array}{r} {\hat{\text{F}}}_{u}={\text{M}}_{m}\frac{{\text{F}}_{u}}{\left\|{\text{F}}_{u}\right\|},\;{\hat{\text{F}}}_{u}\in \left(0,\;\lambda_{m}\right]. \end{array}$$

**Figure 3:**
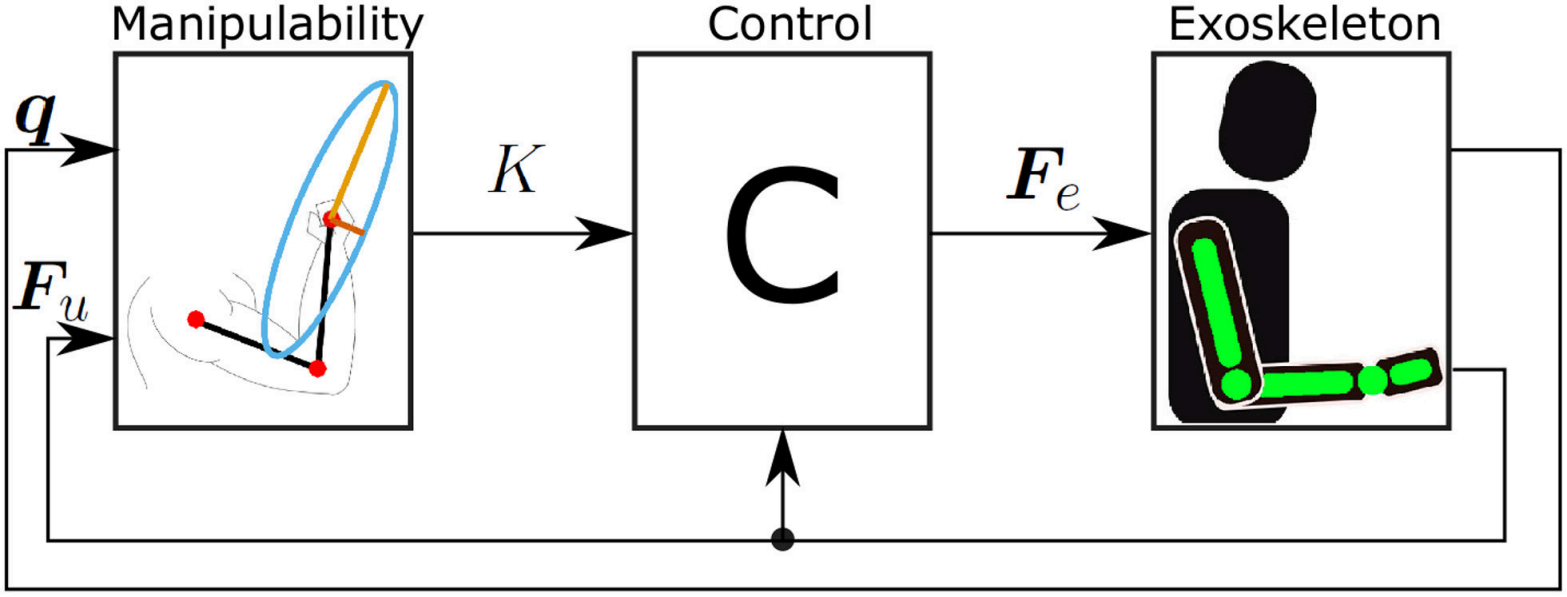
Block diagram of the proposed control concept. The force generated by the user ***F***_*u*_ is used to calculate the desired supporting force of the exoskeleton ***F***_*e*_. This is then used as a force reference for the exoskeleton.

As a result, the exoskeleton provides a supportive force ***F***_*e*_, which is based on the muscular manipulability model in the direction of the user's force. Note that with this approach the exoskeleton provides no supportive force when the direction of the user's force is aligned with the major axis of muscular manipulability ellipse. In this case, the supporting force of the exoskeleton as define in (11) results in ***F***_*e*_ = 0. In all other cases, the exoskeleton will provide a supportive force to compensate for the difference between major manipulability and the manipulability in the direction of movement as illustrated in Figure 1. The block diagram of the control concept is shown in Figure 3.

## 4. Evaluation

### 4.1. Subjects

Nine healthy male subjects participated in this study with an average age of 29.4 years (SD = 2.02 years), weight of 70.8 kg (SD = 2.01 kg) and height of 175.9 cm (SD = 1.29 cm). Prior to their participation, the subjects were informed about experimental procedures, potential risks and the aim of the study. The study and the informed consent signed by subjects was approved by Advanced Telecommunication Research Ethics Committee (Nos. 730, 731).

### 4.2. Experimental Setup

**Figure 4:**
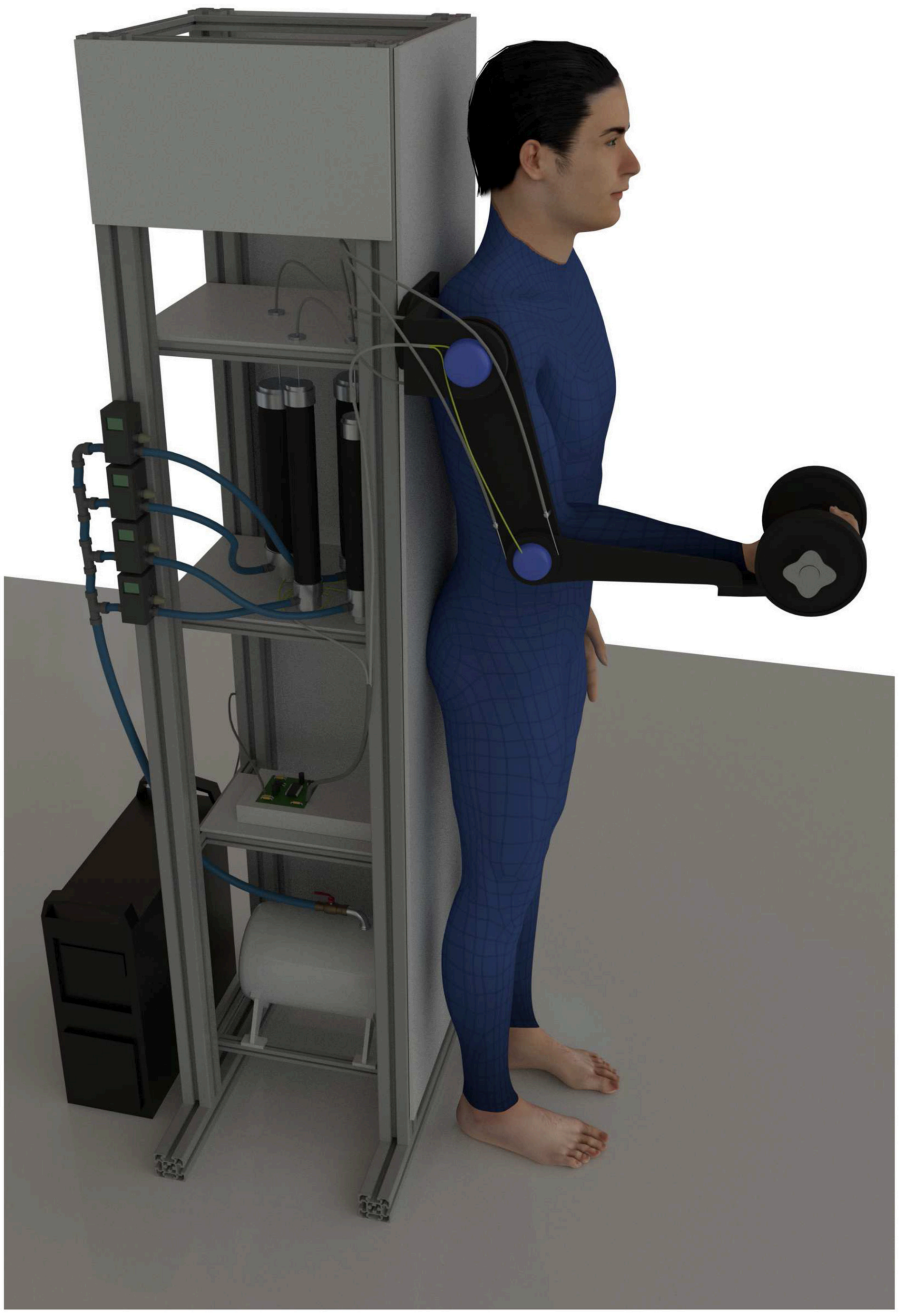
Illustration of experimental setup. The image shows the subject coupled with the exoskeleton where the arm is strapped to the subject arm with straps. The subject is holding a weight in the arm and was instructed to do a periodic lifting motion between designated targets. The lifting period was controlled by a metronome.

The proposed method was evaluated on a pneumatically actuated arm exoskeleton as illustrated in Figure 4. Nevertheless, the proposed method is general and can be used with any exoskeleton that has force/torque sensing capabilities. The exoskeleton was developed at the Department of Brain Robot Interface, ATR, Japan (Noda et al., 2014). For evaluating the manipulability-based assistance, the motion was limited to a sagittal plane and we used only shoulder and elbow joints. The human arm was modeled as a planar two-segment serial mechanism. In this model the first joint represent the shoulder and the second joint represent the elbow. Note that we considered the wrist as a part of the forearm. The arm configuration and endpoint force were measured in real-time by encoders in exoskeleton joints and a force sensor, respectively.

### 4.3. Experimental Protocol

**Figure 5:**
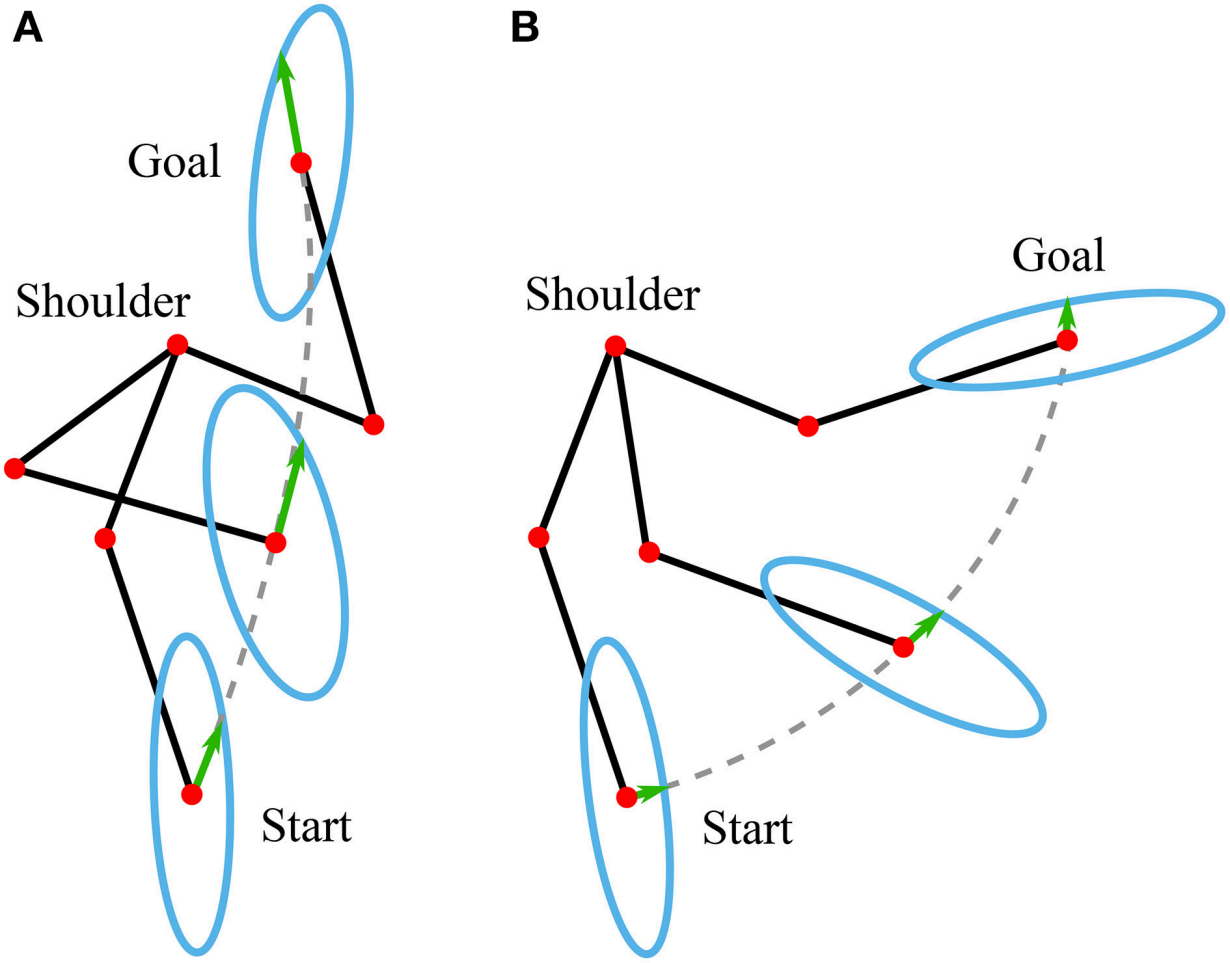
Different configurations of arm and their corresponding muscular manipulability ellipses for the two motions. **(A)** Motion in the high manipulability region **(B)** Motion in the low manipulability region. Green arrows point into the current direction of motion and their size correspond to the current manipulability.

Each subject was wearing the arm exoskeleton and was holding a 4 kg weight in their hands as shown in Figure 4. They were asked to move toward two different targets from the same starting position, at which the posture of the subject's arm was aligned with the body. Both targets, the starting position, the initial arm pose and the final arm pose are illustrated in Figure 5. To accentuate the differences, the motion paths were selected so that the most part of the motion toward the target A is in the area of high muscular manipulability and the motion path toward the target B is mostly in the area of low muscular manipulability. Note that along the path the muscular manipulability characteristics are not constant and the proposed method adaptively assisted the human motion accordingly. The length of the path for both motions was the same and about 70 cm.

The experiment was divided into four sessions:

- High-Unsupported: high manipulability motion without exoskeleton support.
- Low-Unsupported: low manipulability motion without exoskeleton support.
- High-Supported: high manipulability motion with exoskeleton support.
- Low-Supported:low manipulability motion with exoskeleton support.

Note that in the cases without the exoskeleton support we consider that the exoskeleton was only compensating its own mass and did not provide any additional support to the user, and in the cases with the exoskeleton support we consider that the arm exoskeleton was compensating its own mass and at the same time providing an additional support for the user based on the manipulability controller. Each session lasted for about 60 s, which resulted in 20 cycles of motion. The movement period of motion was maintained by asking the subjects to follow the rhythm of a metronome.

### 4.4. Data Processing

In each session, we collected motion data with the sampling frequency of 100 Hz and EMG data with the sampling frequency of 1kHz. The motion variation was assessed by the deviation of movement with respect to the straight line between the starting position and the target position. The deviation was quantified as the unsigned area between the actual movement and the straight line and is denoted as *trajectory area*.

The human effort required to perform the motion was assessed by measuring and analyzing the EMG signals of Biceps long head and Pectoralis minor muscles. These two muscles are among the most dominant arm flexors for the arm motion in the sagittal plane. Each EMG signal was rectified and filtered with a second-order low-pass filter with a cut-off frequency of 3 Hz. To obtain the muscular activation, we normalized the processed EMG signal by the EMG measured during the maximum voluntary contraction of the respective muscle. To quantify the human effort, the processed and normalized EMG signal was integrated over time. From now on, the muscular activity will be denoted as 0 ≤ *EMG* ≤ 1 and its time integral as *iEMG*.

The statistical analysis was performed using Statistics and Machine Learning Toolbox in MATLAB. We calculated average movement times required for the motion, trajectory area, and iEMG during each of the four sessions for each subject. We then used these average values of each subject for statistical analysis. We investigated the effects of the exoskeleton device with the proposed controller on the movement times, movement variations and human effort using two-way repeated-measures ANOVA with independent variables [controller(2) × targets(2)]. The differences between the trajectory areas and the differences between the iEMGs were tested with *post-hoc t*-tests with Bonferroni correction. The level of statistical significance used was .05 for all statistical tests.

### 4.5. Results

Analysis of variance showed no significant effects of the exoskeleton device on the movement times between both high and low manipulability motion [*F*_(1,8)_ = 2.47, *p* = 0.15] and supported and unsupported motion [*F*_(1,8)_ = 1.36, *p* < 0.28]. There was no significant interaction [*F*_(1,8)_ = 0.06, *p* = 0.93] between the effects of low and high manipulability motion, and the supported and unsupported motion on the time to reach the target.

**Figure 6:**
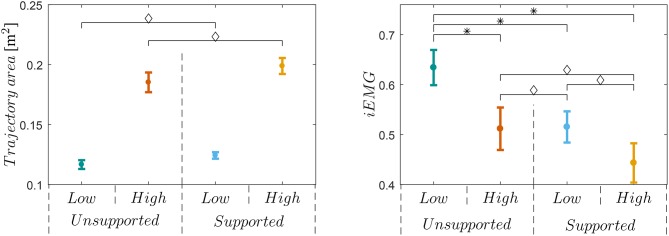
Left plot shows trajectory area for all four sessions and all subjects. Results show that was no significant difference (◇) between sessions with or without exoskeleton support for a low manipulability sessions and for a high manipulability sessions. Right plot shows iEMG for all subjects and sessions. Results show that only low-unsupported motion was significantly different (*) with others. Note that there was no significant difference (◇) between low-supported and high-supported motion.

Analysis of variance showed significant effects of the exoskeleton device on the both high and low manipulability motion [*F*_(1,8)_ = 3.23, *p* = 0.01] and supported and unsupported motion [*F*_(1,8)_ = 108.12, *p* < 0.01] on the trajectory area. There was no significant interaction [*F*_(1,8)_ = 1.26, *p* = 0.29] between the effects of the exoskeleton device on the low and high manipulability motion and supported and unsupported motion on the trajectory area. *Post-hoc t*-tests showed that trajectory area of the *Low-* Unsupported and Supported is statistically different from the trajectory area of the *High-* Unsupported and Supported [*t*_(9)_ = 9.33−12.58, *p* < 0.01]. There is no difference between trajectory areas of Low-Unsupported and Low-Supported, and trajectory areas of High-Unsupported and High-Supported. The left diagram in Figure 6 shows the means and standard errors (SEM) of the trajectory areas for all supported and unsupported motions.

**Figure 7:**
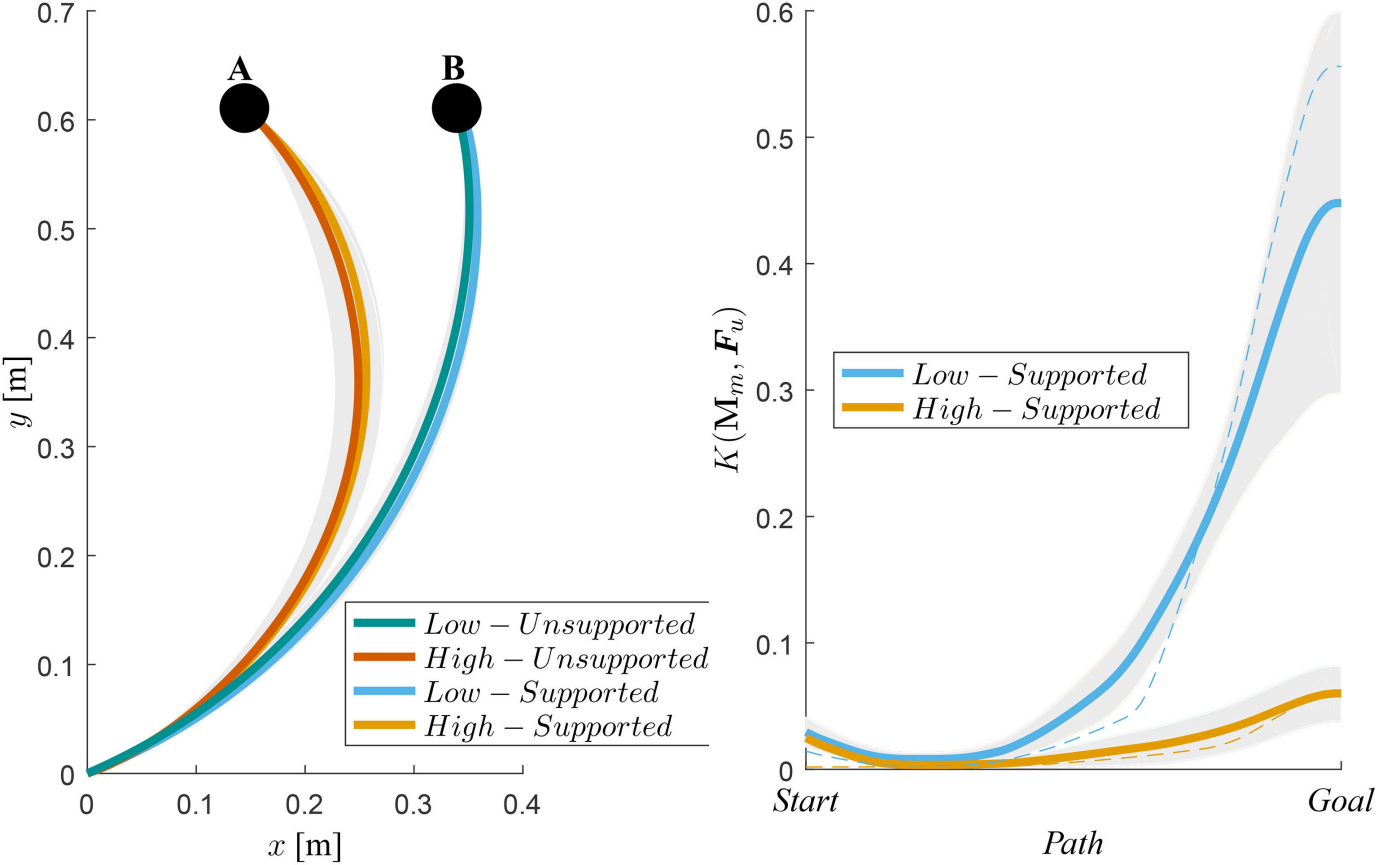
Left-hand side plot shows the average hand trajectories of supported motion (orange) and unsupported motion (blue). A denotes the target for the trajectories during the high-manipulability motion and B denotes the target for the trajectories during the low-manipulability motion. Right-hand side plot shows gain *K*(**M**_*m*_, ***F***_*u*_), i.e., how much the user force ***F***_*u*_ was amplified, from the Start to the Goal position. The shaded area represents standard deviation from the mean of all subjects. As an example, the dotted lines show gain values for one subject.

Average motion paths and their standard deviations are shown in Figure 7, where we can see a negligible difference between the unsupported motion and the supported motion paths. Right plot on Figure 7 shows the gain *K*(**M**_*m*_, ***F***_*u*_) and their standard deviations with respect to the path for High and Low manipulability targets.

Analysis of variance showed significant effects of the exoskeleton device on both high and low manipulability motion [*F*_(1,8)_ = 7.22, *p* = 0.03] and supported and unsupported motion [*F*_(1,8)_ = 48.12, *p* < 0.01] on the iEMG activities. There was no significant interaction [*F*_(1,8)_ = 2.09, *p* = 0.19] between the effects of exoskeleton device on low and high manipulability motion and supported and unsupported motion on the iEMG activities. *Post-hoc t*-tests showed that iEMG activities of the *Low-Unsupported* motion is statistically different from any of the others [*t*_(9)_ = 3.52−5.64, *p* < 0.01]. The right diagram on Figure 6 shows mean values for all supported and unsupported motions.

**Figure 8:**
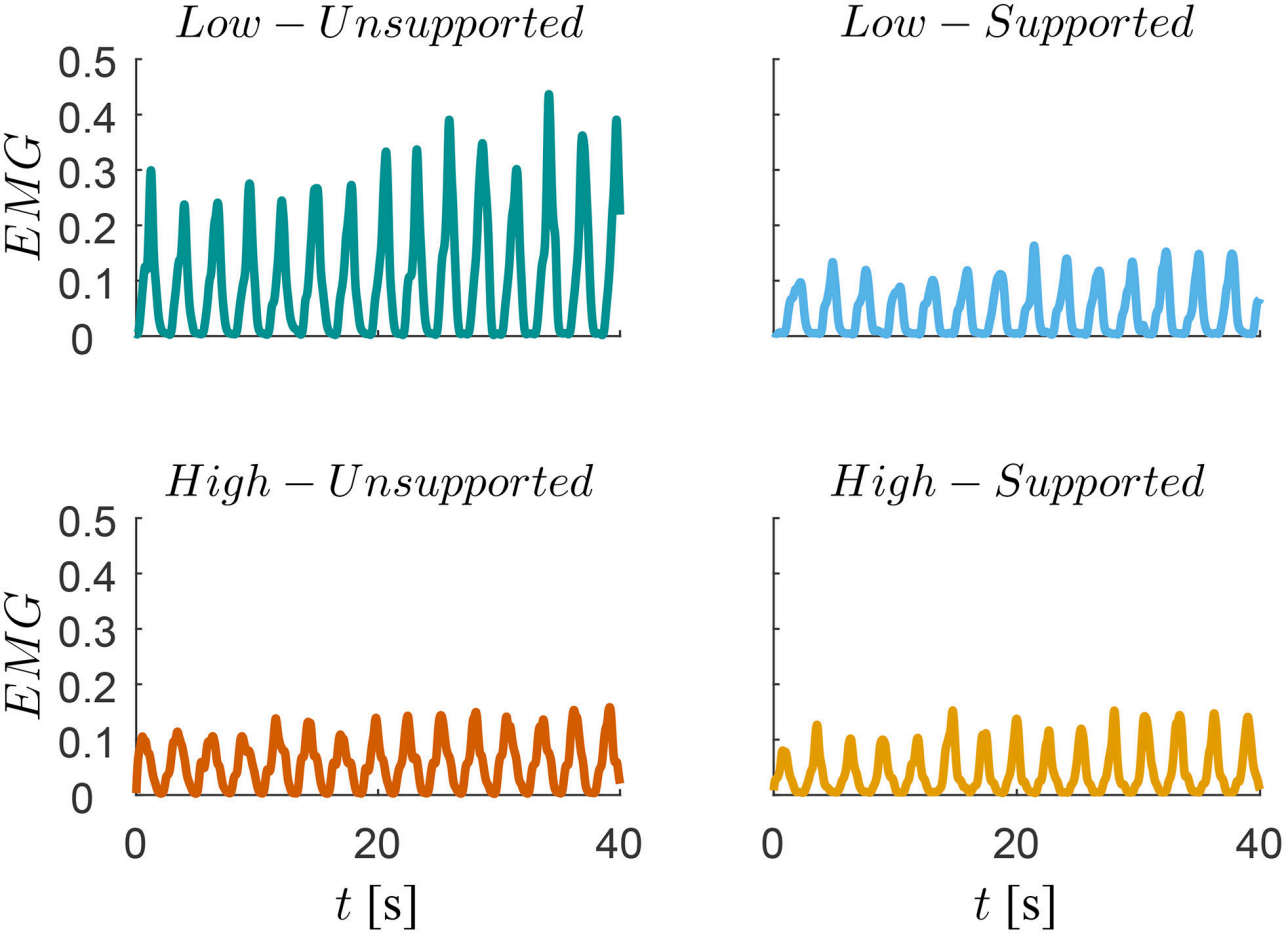
Traces of the muscular activity measured by EMG during the low-manipulability motion (top graphs) and during high-manipulability motion (bottom graphs). Muscular activity during the unsupported motions are represented on the left-side panes while the muscular activity during the supported motions using the proposed controller are represented on the right-side pane.

A trace of the EMG signal for one subject is shown in Figure 8, where we can see a significant reduction of human effort for low-supported motion compared to the low-unsupported motion, but there is no difference between others (high-unsupported, high-supported and low-supported).

## 5. Discussion

The goal of this study was to introduce a novel exoskeleton control approach that selectively augments the performance of the human user. This study also evaluate the impact of the controller on user motion and effort with multi-subject experiments. We hypothesized that the proposed control approach will reduce human effort without diverting from the normal unassisted motion trajectory.

It is evident from the results that by exploiting the anisotropic effect of the controller, the human effort remains similar for the low-supported case compared to the high-supported and high-unsupported case, i.e., there was no significant statistical difference between these three. In effect, the human effort of subjects wearing the exoskeleton device with the proposed method became equal for both motions. On the basis of the results we assume that the approach would generalize for arbitrary motion in the entire workspace.

In addition, it is also evident from Figures 6, 8 that the unsupported motion in the low-manipulability area requires considerably more muscular effort than the unsupported motion in the high-manipulability area. The results of the supported motion indicate that the proposed method was able to effectively reduce the human effort for the motion in the low-manipulability area. Results also showed that the level of the human effort in the low-manipulability area with the proposed controller is comparable to the motion in the high-manipulability area.

By augmenting the human end-point force capabilities considering the instantaneous arm configuration and the direction of motion we showed that a spherical end-point force manipulability can be effectively maintained throughout the entire workspace of the human arm. We also found out that the proposed control approach did not alter user motion trajectory (Figures 6, 7), since the difference between the two was statistically insignificant. This suggests that the method can augment the force manipulability without affecting the normal movement characteristics of the exoskeleton users.

In the analysis we were interested in normal human behavior therefore we used healthy subjects. The goal of the paper was to a design controller aimed at power-augmentation scenarios. Rehabilitation scenarios include disabilities and abnormal human behavior, and were therefore not in the scope of this paper. However, the results obtained within the scope of this paper are the basis for our future research, where we will be interested to see if this method can be applied in rehabilitation scenarios for subjects with disabilities.

The proposed manipulability-based power-augmentation method fundamentally differs from assist-as-need methods usually employed in rehabilitation scenarios. Assist-as-needed controllers (Wang et al., 2010; Pehlivan et al., 2016; Shahbazi et al., 2016; Li et al., 2017; Luo et al., 2019) basically provide an exoskeleton assistance when the user is not able to follow the therapy-based predefined trajectories, i.e., the level of assistance is based the error between the desired motion and the actual motion. On the other hand, the proposed controller employs no predefined trajectories and the user is free to perform the movements as desired, while the the level of assistance is based on the measured manipulability in a given configuration at any given sample time. The main advantage of the assist-as-needed rehabilitation methods is that the the controller can operate with predefined desired trajectories, which is paramount for various therapy programs. The main advantage of the proposed manipulability-based power-augmentation method is that the controller does not take any predefined reference trajectories and therefore the users can define the motion themselves.

The proposed method was tested on two DoF arm exoskeleton that was available to us at the given time. In future, we will develop more complex exoskeletons and test the proposed method on more DoF.

## Author Contributions

TP, LP, JM, and JB contributed to the design, execution, and drafting of this work, and approved the final manuscript. Experimental data was collected by JB. Data was analyzed by TP.

### Conflict of Interest Statement

The authors declare that the research was conducted in the absence of any commercial or financial relationships that could be construed as a potential conflict of interest.
